# The Young Practitioner and Porcelain

**Published:** 1906-03

**Authors:** Geo. B. Mitchell

**Affiliations:** Buffalo, N. Y.


### THE YOUNG PRACTITIONER AND PORCELAIN.

By Geo. B. Mitchell, D. D. S., Buffalo, N. Y.

It may not be inapropos, to say that Bacon's advice about books, "some are to be tasted, others swallowed, and some few to be chewed and digested," is applicable to porcelain work and the beginner, except that the novice in this line should apply all of the above operations *thoroughly* to porcelain work, not only at the start, but at all times during his professional career —for there is no operative procedure which the ethical practitioner is called upon to perform, which requires such keen perception to details, exquisite tact, and thoroughness of procedure, as does this specialty of dentistry. Well might a volume be written on the subject of "porcelain and beginners"

alone—as the advice would reach to us all. With but few exceptions, are we not all novices in this art?

It is not within the province of this short article to elucidate all the points in porcelain technique, but to record a few jottings of major points, as they appear on the mental horizon of the writer, as he passes over the porcelain field.

Beginners, first purchase a copy of Dr. V. Walter Gilbert's *Dental Porcelain*, and *especially*, inwardly digest the article on Laboratory experiments, and if at all possible, perform the same experiments for yourself. Start your experimentation (and make it long and thorough), with the low-fusing bodies, even if you believe the high-fusing the better. This for many reasons, among which are cheapness, ease of manipulation, and difficulty of results to be overcome, makes one familiar with porcelain.

Each manufacturer claims his product "the best," but if one will only experiment fully, he is the one to best judge the relative merits between high-fusing and low-fusing bodies, and to make his choice as adapted to his own personal requirements.

Prepare at least one hundred cavities in the laboratory before you start one in the oral cavity. Remember, to always have a flat seat to your cavity; the walls straight and as nearly parallel as possible. Cavities in which the walls bear an *angular* and not a curved relation to one another and to the base, is the proper cavity preparation.

*Matrices.*—For low-fusing bodies, use Williams' soft gold, Nos. 30 and 40. For high-fusing bodies use Rowans platinum 1/1000 or 1/1200. If a low-fusing body is to be used in large contour work, it is advisable to use a platinum matrix, thereby facilitating retrial and reburnishing at any stage of the operation and permitting you to obtain a better idea as to the progress of contour and extent desired.

In introducing matrix to the cavity, try wet cotton instead of spunk, etc. It will work easier and with less liability to tear the matrix. As soon as you have obtained the matrix to the floor entire, take a strip of tape or baby-ribbon, a trifle wider than the length of the matrix, and draw it down and across, so the platinum will lap all margins. Finish burnishing, re-annealing often, eradicating all wrinkles,—then take a strip of rubber-dam, as per tape, draw tightly over matrix, grasp ends and burnish matrix all over—producing perfect adaptation and no rocking (Dr. Reeves original procedure). Pack matrix full with pure Japanese camphor, not American camphor, which is too brittle, remove and invest. With but few exceptions, as in extensive contouring, the writer always invests the platinum matrix, thereby improving adaptation by fifty per cent. This, of course, is not obligatory, when using platinum.

Procure several pencils with rubber tips and trim to forms needed for work in adapting matrix to cavity. In the manipulation of the porcelain body, use red sable brushes.

As regards working on impressions of the cavity, produced by dental lac, etc., there is but this to say:—when you burnish your matrix to the tooth in the mouth, you are working on *the* original model, "nuff said."

*Study* well and long, while at other operations, the underlying colors of the teeth *in the mouth*, as all extracted teeth are of the same color. Obtain the color scheme for each operation by baking in layers, not by mixing two or more colors, as this will often give you a color far from that which you expected. *Choose your colors, with tooth wet by saliva.* Use the whitest body you can procure to line the matrix, in first baking. This almost solves the cement question. Go to the library and study diligently a good authority on colors and color schemes and Tyndall on "Light;" experiment with a box of water-colors, mixing the basal colors with one another and you will be surprised by the benefit you will receive.

Mix your cement with water to try in the inlay—until you strike the proper color.

Undercut your cavity previous to cementation, the same as you would were you to insert a permanent cement filling. Triturate the cement powder in a mortar to an extremely fine consistency before mixing. The finer the grit, the better the cementation (Head). Always groove the underpart of the inlay. If possible, make the grooves in diverging directions, thereby aiding retention.

Etch bottom of inlay with the white acid and *be sure* when dry to use a stiff brush to remove the dust produced by the acid on the etched part of the inlay.

Wedge the inlay to place by wooden wedge and tie a ligature tightly around tooth and inlay two or three times before dismissing patient; allow it to remain 24 hours. You will then not have the inlay raised at the margins at the next sitting, if you do so. Expansion of cement after crystallization is the cause of this and we know all cements expand.

Allow, *at least,* one-half hour for the setting of the cement. Here is where many an operator fails, in what would otherwise have been a perfect operation. Harvard Cement is considered by most porcelain operators to be the most satisfactory in results.

Confine your first work in the oral cavity strictly to labial cavities; and until you have obtained good results, rectified by another practitioner, do not feel assured that you have mastered porcelain technique, in the least. Read all the articles on porcelains, cements, colors, etc., you can find. Never try an experiment in the mouth—try a result. Confine experiments to the laboratory. Do not attempt porcelain crown work until you are thoroughly acquainted with the very feminine material—porcelain. Don't become discouraged. Above all be conservative. Let the *best* be your only product, and when you become efficient, do not plebeianize the beautiful results obtained in porcelain operations by cheap prices.

